# Dr. Vincent A. Baker

**Published:** 1914-06

**Authors:** 


Dr. Vincent A. Baker, Syracuse, 1854; Eclectic Medical College of the City of New York, 1873; died at his home in Adrian, Mich., March 20, aged 82.

